# Metal-Based Compounds in Antiviral Therapy

**DOI:** 10.3390/biom12070933

**Published:** 2022-07-03

**Authors:** Chiara Abate, Federica Carnamucio, Ottavia Giuffrè, Claudia Foti

**Affiliations:** Department of Chemical, Biological, Pharmaceutical and Environmental Sciences, University of Messina, Viale Ferdinando Stagno d’Alcontres 31, 98166 Messina, Italy; chiara.abate@unime.it (C.A.); carnamuciof@unime.it (F.C.); cfoti@unime.it (C.F.)

**Keywords:** antiviral drugs, drug delivery, metal compounds, metal nanoparticles, vaccines

## Abstract

In recent years, the study of metal complexes and metal-based nanomaterials has aroused particular interest, leading to the promotion of new effective systems for the abatement of various viral diseases. Starting from the analysis of chemical properties, this review focuses on the employment of metal-based nanoparticles as antiviral drugs and how this interaction leads to a substantial enhancement in antiviral activity. The use of metal-based antiviral drugs has also spread for the formulation of antiviral vaccines, thanks especially to the remarkable adjuvant activities of some of the metal complexes. In particular, the small size and inert nature of Au- and Ag-based nanoparticles have been exploited for the design of systems for antiviral drug delivery, leading to the development of specific and safe therapies that lead to a decrease in side effects.

## 1. Introduction

As it is well known, metal ions are important components of life and are involved in many vital functions in biological systems [1,2]. Just to mention a few, they are essential cofactors of many enzymes and contribute to regulate the electrolyte balance, electron transfer, and transport of oxygen. They are also implicated in regulating both the immune system and host defense against invading pathogens. The broad spectrum of properties of metals, such as different coordination numbers, geometries, redox states, but also reactivity and thermodynamic behavior, has determined their important role in medicine and extensive use in the treatment and diagnosis of many diseases.

Throughout history, as far back as ancient Egypt, Greece, and Rome, as well as in the ancient Aztec civilization, many metals and metal compounds have been used for the treatment of disease. Copper has been used as a sterilization agent for drinking water but also for headaches and for leg ulcers that are associated with varicose veins, zinc as an antiseptic, mercurous chloride as a diuretic, and arsenic compounds for the treatment of syphilis. In 1965, the discovery by Rosemberg [3] of cisplatinum as an anticancer drug led to the development of numerous metallic drugs and many metal compounds have been designed and explored for both therapy and diagnosis [4].

Recently, continuous advances in the nanotechnology sector have led to significant progress in the diagnosis but also in the prevention and pharmacological treatment of many diseases [5,6,7]. The use of nanomaterials has made it possible to overcome some of the problems related to traditional metallodrugs, such as the poor solubility, toxicity, bioavailability, and stability of drugs, increasing at the same time the selectivity for viruses and efficacy.

In the last two years, the pandemic caused by the coronavirus SARS-CoV-2, that reached more than 50 million official cases, has focused the attention of many researchers on the study of systems useful for diagnostic and curative purposes as well as vaccines. Although the attention towards this virus has globally dominated the research in the last period, we must also remember the numerous studies that were conducted to fight other important viruses such as Dengue (DNV), Zika (ZIKV), Chikungunya (CHIKV), human immunodeficiency (HIV), Herpes simplex (HSV), Hepatitis C virus (HCV), yellow fever, Measles viruses, and also *Influenza* A (e.g., H1N1, H5N1).

**Figure 1:**
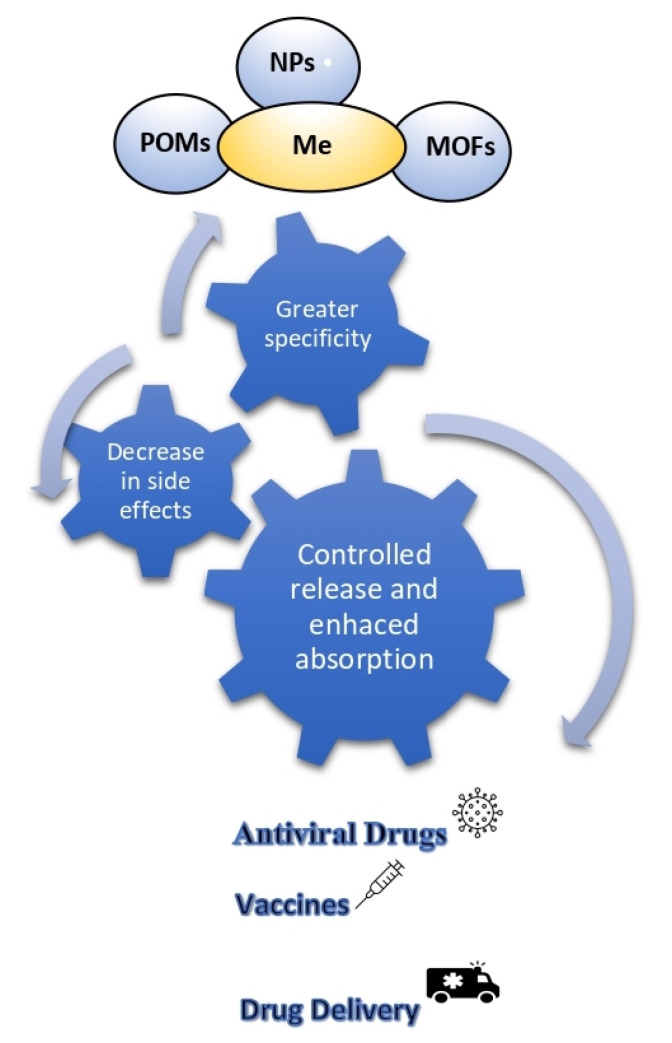
Schematic representation of the main properties and applications of metal-based compounds.

Metal compounds play an important role acting in many ways: they can interrupt infections, alleviate symptoms, regulate the human immune system, inhibit critical enzymes in the process of virus replication, or as act adjuvants to enhance the efficacy of antiviral drugs and vaccines [8,9], as shown in Figure 1.

In this review we report an analysis of the recent progress in the use of metal compounds as antiviral drugs, starting from the study of the chemical properties of these metal compounds with particular attention to thermodynamic properties, speciation, and structural characteristics. To give a complete picture of the importance of metal complexes in combating viruses, attention was paid not only on the most recent advances of metal complexes as antiviral drugs, but also on their use in vaccines and drug delivery systems.

## 2. Chemical Properties of Metal Drugs

Antiviral activity of metal complex species could be affected, first of all, by their own structural characteristics, speciation, nature of metal ions, as well as by the free functional groups in the ligands. These moieties are not only involved in the recognition processes, but also improve the solubility and effectiveness of the complexes. On the other hand, the selective recognition and interactions of a ligand towards biomolecules also depend on the size and nature of its substituents and molecular structure, which affect the lipophilicity, stereochemistry, kinetic, and thermodynamic stability [10].

Particular attention must be paid to the thermodynamic properties, such as acid-base behavior and solubility in aqueous solutions. Drug molecules generally contain one or more ionizable sites. The knowledge of the dissociation constants of pharmacologically active molecules is necessary to define the state of ionization which influences their characteristics, such as aqueous solubility, permeability, partition coefficients, and pharmacokinetic properties [11]. The solubility of a drug is a key factor for purification processes, pharmaceutical development, crystal form screening, and production control.

Another important property is the lipophilicity that enhances the permeability through the lipid layers of the cell membrane. The coordination of compounds with metal ions influences the lipophilicity, as it decreases the polarity of the ions due to the partial sharing of the metal ion with the donor groups of the compound [12,13].

Furthermore, it is known that often the complexation with metal ions enhances the antiviral activities of drugs [14], in concordance with the importance of metal ions in biological systems [15]. More precisely, many compounds employed as drugs exhibit modified pharmacological and toxicological profiles when they are administered in their metal-based form. For these reasons, in the literature there are several papers on the metal ion complexation of antiviral drugs, such as valacyclovir, acylhydrazones, ribavirin active against HCV, 2-hydroxybenzamides-base compounds used as anti-influenza virus, adefovir, biguanide derivatives active against herpes, cidofovir employed for the treatment of cytomegalovirus retinitis, and polyoxovanadates.

In particular, the protonation constants of valacyclovir (L-valine, 2-[(2-amino-1,6-dihydro-6-oxo-9-H-purin-9-yl)methoxy]ethyl ester), an antiviral molecule used against HSV, was reported. The complexation behavior of valacyclovir with Cu(II) was also investigated, evidencing the formation of 2:1 and 2:2 complexes. The techniques that were employed were potentiometry, UV-Vis, IR, MS, magneto-chemical, thermogravimetric, atomic absorption, conductivity, and elemental analysis data [16]. The biological activity of valacyclovir and its Cu(II) complexes have also been investigated, and the results highlighted the complexation with the metal ion enhances the biological activity. This can be explained by the chelation theory, i.e., the polarity of the metal ion decreases due to the partial sharing of the charge with the donor groups and to the delocalization of the π electron on the chelated ring. In this way, the metal ion lipophilic character increases, favoring its permanence through the lipid layer of the cell membrane [16].

A recent paper reported the acid-base and coordinating behavior towards Cu(II), Mn(II) and Mg(II) of three 2-hydroxy-3-methoxyphenyl acylhydrazones (HL_1_, HL_2_, and H_2_L_3_), that show activities against HSV and vaccinia virus [17]. A potentiometric and microcalorimetric study was conducted to reveal the behavior of the three acylhydrazones compounds in a mixed solvent methanol/water = 9/1. The metal complexing abilities of acylhydrazones are responsible for the biological activity of these compounds. The results proved the coordinating behavior of acylhydrazone molecules towards Mg(II), Mn(II), and Cu(II) is different depending on the R group. The three ligands, and in particular H_2_L_3_, showed a greater complexing capacity towards Cu(II). A significant correlation between the high complexing capacity and the good antiviral activity against HSV and vaccinia virus (EC50 ~ 1.5 µM, minimal cytotoxic concentration = 60 µM, selectivity index = 40) was evidenced and should be further investigated [17].

The complexation of molecules that are employed against HCV, such as ribavirin (1,β-D-ribofuranosyl-1,2,4-triazole-3-carboxamide, RBV), was also investigated. In more detail, complexes of RBV with Cu(II),whose hepatic and serum concentration significantly increases due to a HCV infection, were studied [18]. The coordination, elucidated by spectroscopic studies and DFT calculations, occurs via triazole N4 and amide oxygen atoms [18].

A total of three synthetized 2-hydroxybenzamides-based compounds, that are potentially used as anti-influenza virus therapeutics, were investigated in terms of acid-base behavior, complexing ability towards Mg(II), and biological characteristics. It was proven that all three ligands (indicated as HL^1^, H_2_L^2^, and HL^3^) are able to bind Mg(II) with different coordination abilities. Potentiometric investigations evidenced that, under physiological pH, the H_2_L^2^ form is the most efficient [19].

For the acyclic nucleoside phosphonate 9-[2-(phosphonomethoxy)ethyl]guanine (PMEG), active as anticancer and antiviral drug, the acidity constants as well as the stability constants of the Cu(II) complexes were obtained by potentiometric titrations in aqueous solutions at t = 25 °C, I = 0.1 M in NaNO_3_ [15].

The dissociation constants of adefovir (PMEA, 9-[2-(phosphonomethoxy)ethyl]adenine) and cidofovir (HPMPC, phosphoric acid) were investigated by potentiometric titrations in NaCl under different experimental conditions [20]. The solvent effects on the dissociation constants of the ligands were also studied as increasing of the ethyl alcohol percentage. PMEA is an active compound against a series of viruses including herpes. HPMPC is employed in AIDS patients for the treatment of cytomegalovirus retinitis [2]. Stability constants of complex species of PMEA and HPMPC with Cu(II), Ni(II), Zn(II), Co(II), Ca(II), and Mg(II) were also obtained [20].

Several complexes between biguanide derivatives NRR’(NH)CNHC(NH)NH_2_ ((RR’bigH: R, R’ = Bu^i^, H; Pr, H; -CH_2_CH_2_OCH_2_CH_2_-) and Co(II) or Co(III) ions were obtained and characterized by X-ray diffraction analysis. Both compounds were active against the second type of virus herpes [21].

A very interesting correlation between the structural stability of metal drugs and their biological activity was reported in the paper of Sigel et al. [22]. Protonation constants of 9-[2-(2-phosphonoethoxy)ethyl]adenine, PEEA and of (2-phosphonoethoxy)ethane, PEE, and the stability constants of the complexes with different divalent cations were determined. Cations under study were Mg(II), Ca(II), Sr(II), Ba(II), Mn(II), Co(II), Ni(II), Cu(II), Zn(II), and Cd(II). The results showed that some of these species form six-membered chelates. Comparisons with complexes that are formed by PMEA (9-[2-(phosphonomethoxy)ethyl]adenine) with divalent cations revealed that five-membered chelates are considerably more stable than the corresponding six-membered ones. This evidence provides an explanation for the excellent antiviral properties of PMEA as opposed to PEEA [22].

Finally, polyoxovanadates (POVs), compounds with anticancer, antimicrobial, and antiviral properties, have been investigated with different techniques, as potentiometry, ^51^V NMR, FT-IR, EPR, and UV-Vis spectroscopy as well as X-ray crystallography. The speciation model referring to vanadate solutions was obtained by ^51^V NMR and potentiometric investigations. It indicates, at pH > 6, the presence of polymers containing from one to five vanadium centers, characterized by individual protonation steps. These species, as tetramer or dimer, are in rapid equilibria and can present selective inhibition of enzyme activities [23].

Finally, another important propriety to investigate for antiviral drugs is the polymorphism, i.e., the ability of a solid compound to present more than one crystal structure [24]. Each polymorph shows different physicochemical properties, such as solubility, melting point, and density, resulting in a significant influence on the efficiency of the drug produced. However, to our knowledge, in the literature there are no reports on this property inherent antiviral metal drugs.

## 3. Antiviral Metal-Based Drugs

As underlined in the Introduction, to improve and/or boost antiviral actions of biologically active molecules, one of the best and smartest strategies was the study of their coordination with metal ions [25]. This topic has attracted the curiosity of the scientific community since ancient times for their peculiar and unique properties. As stated above, the chemical properties and reactivity of the metal-based complexes are affected by the electronic structure, oxidation state and redox potential of the metal ions, nature of the ligands, as well as their coordination sphere. In turn, these properties influence the biological activity that can be exerted in many ways such as direct inhibition of enzymes, alterations of transcription factors, interaction with a variety of biomolecules through coordinative bonding, enhanced lipophilicity, alteration of cell membrane functions, and interference with the cell cycle. By virtue of these characteristics, metal complexes have emerged as promising tools for medicinal application [26,27]. In the literature, there are reported a very high number of studies concerning their synthesis, antiviral properties, and possible mechanism of action against HIV, Ebola, Influenza, and SARS viruses. Between 2019 and 2022, many reviews have been published that are focused on different aspects and characteristics of metal complexes as antiviral agents [4,5,7,8,13,28,29]. Table 1 summarizes the main antiviral metal complexes and their applications in antiviral therapy.

In particular, in-depth analysis of the antiviral complexes of first, second, and third-row metals is schematized in [8], where the molecular targets implicated in the antiviral activities are also discussed. Transition metal-based drugs, by virtue of their above-mentioned properties, give a better antiviral response than the potent post-infection therapies with favipiravir, remdesivir, lopinavir, and tocilizumab [27].

Particular attention should be paid to promising antiviral metal-complexes with a soft metal center, such as gold(I), platinum(II), and silver(I), that are endowed by a great affinity for free, accessible, and functionally-important thiol or selenol groups of target proteins and enzymes [26]. Since antiviral platinum compounds have been widely described by De Castro et al. [30], this review focuses on the gold and silver ones. About that, the gold(I) drug Auranofin (AF), [2,3,4,6-tetra-o-acetyl-L-thio-β-D-glycol-pyranoses-S-(triethyl-phosphine)-gold(I)] (brand name is Ridaura^®^), is noteworthy for its remarkable antiviral and anti-inflammatory properties, and its pharmacological actions [8,26,31]. Although its mechanism of action is still unclear, the orally administered antiretroviral AF was active against HIV. A recent study has proven that AF is also active in inhibiting SARS-CoV-2 replication in vitro [32,33]. Other gold drugs, such as aurothiomalate and aurothioglucose, or the commercially available AF analogue, chloro-(triethylphosphine)gold(I), AF-Cl [26], could be also used as promising antiviral agents. Singh et al. [34] have reported a study on the antioxidant, antimicrobial, antiviral, anticancer, anti-inflammatory, and enzyme inhibitor properties of pyridyl-based organochalcogen ligands and its metal complexes. Among all, 7-Azabenzisoselenazol-3 (2H)- compounds exhibit antiviral activity against HSV-1 virus (within a range of 0.4–2.0 µg/mL) and the organoselenium Ebselen was found efficient in the treatment of SARS-CoV-2-infected Vero cells.

Metal complexes with Schiff bases are widely discussed in the reviews [28,35,36,37], and here are only briefly mentioned. Indeed, the Schiff bases are proposed as ideal candidates for coordinating the transition metal ions and preparing different enriching metal complexes with various anticancer, antibacterial, and antiviral properties. They are endowed with efficient chelating frameworks and flexi-dentate properties; in particular, soft ligands ease the synthesis, modifications, and substitutions of their structure and, therefore, flexible chains [38]. By way of an example, a Schiff base-cobalt(III) complex, namely Doxovir, was found to be efficient against drug-resistant HSV type I [39].

In addition to the Schiff bases, coordination polymers (CPs) and metal-organic frameworks (MOFs) [37,40] have also sparked increasing and recent interest in their ability to coordinate many metals. In the above-mentioned review [37], non-toxic Fe(III)-MOFs are debated as nanocarriers and molecular sponges for azidothymidine triphosphate (AZT-TP), and are designed for the encapsulation and controlled release of subunit vaccines to improve the cellular immune response by stimulation of cytotoxic T lymphocyte (CTL) production. As well as the Zn(II)-MOFs, or else the Ag(I)-MOF, is noteworthy for its antiviral activity toward human adenovirus 36 (HAdV-36) [41]. On the other hand, although further studies are still needed in this area, the antiviral properties and metal complexes with phytochemicals, such as phenolic compounds and their derivatives, are widely discussed in [13]. For example, the Ga(III)-curcumin complex has displayed considerable antiviral effects against HSV-1, and Cu(II)-curcumin complexes have shown an effective antiviral response against vesicular stomatitis virus (VSV), coxsackie virus B4, and respiratory syncytial virus (RSV) [42].

Special attention should be paid to metal-based nanomaterials. With regard to this, in [5], the authors have analyzed the wide antiviral activity of gold and silver nanoparticles and also that of metal oxide nanoparticles, such as zinc oxide nanoparticles. The latter and similar metal oxide nanoparticles, CuO, SiO_2_, TiO_2_, and CeO_2_, have exhibited a potent response against influenza viruses (H3N2 and H1N1), HBV, HSV, HIV-1, HSV, dengue virus type-2, foot-and-mouth disease (FMD) virus, and (VSV) [43]. Transition metal-based nanomaterials, by virtue of their high specific surface area and small particle sizes, benefit from virus-inactivating ability, interacting with viral surface proteins or their genetic materials by Kazimir and van der Walls forces. Yang et al. [7] have summarized the antiviral properties of metal-based nanoparticles, such as Au, Ag, CuI, and metal oxide nanoparticles, such as TiO_2_, ZnO, and Fe_3_O_4_, depending on their three inhibition pathways of action. Tortella et al. [29] emphasize the improvement in the use of silver (AgNPs) and copper (CuNPs) nanoparticles as antiviral agents, as well as against SARS-CoV-2. Their antiviral activity, demonstrated in vivo and in vitro, has a broad range of action acting against Influenza A, HSV type 1 and 2 (HSV-1 and HSV-2), Hepatitis A virus (HAV-10) and Hepatitis C, coxsackie B4 virus (CoxB4), HIV, RSV, and SARS-CoV-2. The authors also described the use of copper-oxide nanoparticles (CuONPs) against the hepatitis C and HSV-1. In a review, an interesting analysis on a Cu(I)- and Cu(II)-based nanocluster (Cu_x_O), Cu_x_O/TiO_2_, is reported [44], together with its antiviral and visible light-sensitive photocatalyst role.

Despite all these reviews that are very accurate, the recent pandemic has led to a proliferation of research in this area and, therefore, further analysis on anti-COVID metal complexes that are reported in the last years is needed. With regard to that, gold(I), bismuth(III) and antimony(III) compounds could be optimal candidates in inhibiting SARS-CoV-2 viral cysteine proteases and its catalytic activities, and in contrasting very effectively virus replication due to their relatively safe toxicity profiles and high affinity with soft ligands. Similarly, few clinically approved bismuth compounds [45], such as the bismuth potassium citrate, ranitidine bismuth citrate, and bismuth citrate, showing peculiar reactivity properties and strongly thiophilic character together with a low toxicity profile, should be included in this kind of evaluation, as well as ruthenium and antimony compounds [26,46]. However, ruthenium compounds preferentially bind the solvent exposed imidazole residues, blocking functionally relevant histidine residues [26]. Speaking of the development of metal-based drugs as anti-COVID agents, two pivotal strategies have been proposed: drug repurposing and new drug discovery. This latter is absolutely more complex and time consuming but might offer greater opportunities. Metallic-nanoparticles, namely silver, copper, and titanium dioxide nanoparticles, have been proposed as alternatives due to their inherent broad range antiviral activities, versatility, persistence, and ability to be effective at much lower dosage. Moreover, nanoparticles are endowed with an ease of surface modification and nanomaterials with magnetic properties can be decorated with specific receptors of the virus, allowing their magnetic extraction using an external magnetic field and a more accurate detection of the virus by means of nanomaterial-based detection [47].

## 4. Metal Complexes in Antiviral Vaccines

Increased knowledge of the mechanism that triggers the immune response has made it possible to optimize the design of new antiviral vaccines that are aimed to obtain greater efficacy and long-lasting immune response [62]. Among the various vaccine therapies that are proposed, metal complexes have aroused particular interest due to their chemical properties and capacity to stimulate the immune system. In Table 1, the main antiviral metal-based compounds are summarized. Mohamed et al. [48] reported a study on the use of tenoxicam-based ternary complexes (H_2_Ten) that were chelated with different metal cations of biological interests, Cu(II), Ni(II), Co(II), Mn(II), Zn(II), Fe(III), and Cr(III), in presence of valine (Val) for the formulation of Infectious Bovine Rhinotracheitis (IBR) vaccine. The synthesized ternary complexes, that were obtained in the molar ratio (1:1:1) (M:H_2_Ten:Val), were analyzed by potentiometric measurements, determining the thermodynamic parameters ΔH and TΔS, needful to define the type of interaction. The humeral immune response of ternary chelates with the IBR vaccine in calves that were vaccinated through serum neutralizations test (SNT), was also evaluated. The results underlined how the presence of metal chelates with the IBR-inactivated vaccine significantly increased its immune activity and, in particular, the use of the inactivated IBR vaccine with Fe(III) and Cu(II) complexes allowed to obtain early and long-term protective immune responses.

A series of polymeric complexes that were based on 5,5′-[3-diyl)]bis(quinolin-8-ole) (H_2_L) with Co(II), Ni(II), Cu(II), Zn(II), and Cr(III) as vaccine therapy against bovine respiratory syncytial (BRS) was synthesized by Sonbati et al. [49]. A comparison between the immune response given by the BRS vaccine and the polymer complexes synthesized was performed through SNT in calves. The administration of the BRS vaccine attenuated with the polymeric complexes and showed high efficacy as it does not induce side effects and allowed to obtain higher immune responses from the second week and for a longer time.

Metallic nanoparticles (MeNPs) have aroused particular interest as adjuvant agents due to their capacity for cell recruitment, ability to increase cytokine production, and induce a humoral immune response [63,64,65,66,67]. Low toxicity, chemical inertness, biocompatibility, and biodistribution properties have made metallic nanoparticles, in particular gold nanoparticles (AuNPs), excellent candidates for the development of nanovaccines during last years. A very recent review by Sengupta et al. [50], shows the role of AuNPs for the formulation of nanovaccines against various infectious agents, including viral agents. Teng at al. [68] exploited the adjuvant capacities of AuNPs for the formulation of FMD vaccines. The use of AuNPs combined with FMD virus-like particles (VLPs) as antigens, usually present in vaccine therapies, has led to an increase in the immune response, given by higher activation of macrophages.

Niikura et al. [51] synthetized gold nanoparticles as adjuvant agents coated with a West Nile Virus (WNV) envelope (E) protein. There were three types of AuNPs in different shape and size that were synthetized and the effects that these properties could have on the production of MNV antibodies were evaluated. A method to increase the effectiveness of vaccines against WNV was also investigated by Fischer et al. [69]. They used nanolipoprotein particles (NiNLPs), formed by phospholipid and lipoprotein bilayers, containing lipid-heads that were capable of chelating nickel ions during the assembly process. A variant form of the WNV envelope protein functionalized with polyhistidine residues (trE-His) was immobilized on NiNLP. The immune reaction produced by a single inoculation of NiNLP-trE-His proved to be superior to the effect given by trE-His alone.

Starorerov et al. [52] investigated the response given by the administration of gold nanoparticles that were functionalized with the enteropathogenic coronavirus of transmissible porcine gastroenteritis (STG) to mice and rabbits. The comparison of the results obtained by administering AuNPs that were conjugated with the STG antigen with those with the STG virus alone showed a higher concentration of IFN-γ in plasma, one of the main inflammatory mediators in viral infections. The authors hypothesized that the dual task of AuNps acting both as carriers and adjuvants, stimulated the phagocytic activity of macrophages and modulated the functioning of T-lymphocytes, causing a marked increase in the immune response.

The development of new vaccine therapies has been of fundamental importance in recent years due to the emergence of SARS-CoV-2, and functionalized metal nanoparticles have aroused considerable interest in addressing this challenge. Sarkar et al. [70] reported an overview on various metal nanoparticles, including gold nanoparticles, silver nanoparticles, zinc oxide nanoparticles, and copper nanoparticles, characterized by high antiviral activity against coronavirus infection and more.

Farfán-Castro et al. [53] used AuNPs functionalized with SH-PEG-NH2 and glutaraldehyde as a cross-linker to anchor a synthetic peptide (S461-493) that was analogous to the B-cell epitope of the SARS-CoV-2 spike protein. The immune response generated by the synthesized AuNP-PEG-S461-493 system appears to be higher than that which was produced by the S461-493 peptide alone, showing a greater production of cytokines. This study highlights how the observed results are comparable to those that were obtained using strong adjuvants.

The ability of AuNPs to increase cytokine production was investigated by Sekimukai et al. [54]. AuNPs were administered as an adjuvant linked to coronavirus spike (S) protein. The study demonstrates that the administration of protein S adjuvanted with AuNPs generates strong IgG responses, but it is unable to induce an adequate immune response for eosinophilic infiltration, a pulmonary immunopathology that occurs after infection. The explanation for the inability to induce protective immune responses could be given a structural modification of protein S, induced by interaction with AuNPs. The secondary structure of the protein is in fact strongly affected by the surface charge present on AuNPs, and a small change in the structure of protein S could negatively or positively affect its immune response.

## 5. Metal-Based Nanomaterials for Antiviral Drug Delivery

The development of nanotechnology has led to significant innovations in pharmaceutical field, including the formulation of nano-systems for drug delivery. Metal-based nanomaterials, for which many examples are reported in Table 1, have been widely considered due to their unique physical and chemical properties as drug vehicles, which have shown numerous advantages, including a greater control of drug release at the action site and specificity, an increase of drug absorption, and a decrease of side effects [6,71,72].

Nanoparticles of noble metals, in particular gold and silver NPs, have been extensively used for the controlled release of drugs for viral infections [73]. Gold nanoparticles (AuNP) are particularly interesting for their small size that facilitate cellular uptake, for their inert nature, and multivalence that allows for the conjugation and simultaneous transport of several drugs [7]. Garrido et al. [55] studied the antiviral activity of gold nanoparticles conjugated to raltegravir (RAL) molecules for the treatment of HIV, and their ability to cross the blood-brain barrier. In order to maximize drug conjugation with AuNP, RAL was functionalized with thiol groups. AuNP-RAL complexes were synthesized in different ratios, 4:1.33:1 and 60:1 (RAL:AuNP) and HIV inhibition was tested in peripheral blood mononuclear cells (PBMCs), macrophages, and human brain micro-endothelial cells (HBMECs). After five days, the results obtained by administering four molecules of RAL with AuNP showed a 25% decrease in HIV-1 replication, while the antiviral response was significantly decreased by increasing the conjugated RAL molecules. The antiviral activity of AuNP alone was also investigated, but this did not show significant results, demonstrating that the antiviral activity given by AuNP-RAL system is mainly provided by the addition of RAL to the metal core.

One of the main objectives of the formulation of nanocarriers is the development of non-toxic systems that not only allow greater efficacy of the drug delivered, but also a decrease of its toxicity. In this perspective, Horcajada et al. [59] developed Iron(III)-based MOFs as nanocarriers capable of improving the administration of antiviral drugs against the AIDS disease. Among the advantages of porous iron carboxylate nanoMOFs, the most important are certainly the non-toxicity and biocompatibility, but also their versatility given by their ability to act as sponges, trapping drugs with different polarities. The properties and transversal capacities of AuNPs have led their widespread use as vaccine carriers. Chen et al. [56] investigated the efficacy of AuNPs as a vector for the peptide vaccine against FMD. AuNPs, ranging in diameter from 2 to 50 nm (2, 5, 8, 12, 17, 37, and 50 nm), have been conjugated to a synthetic peptide-like FMD virus protein. The functionalization with a residue cysteine at the C-terminus allowed to obtain the maximum conjugation of the protein to AuNPs. The more satisfactory results obtained by administering pFMDV-AuNPs in the size range 8–17 nm, induced immune responses that were three times higher than those obtained with pFMDV-keyhole limpet hemocyanin (pFMDV-KLH). The activity of AuNPs alone was also investigated but they did not show any meaningful antibody response. Yan et al. [60] synthesized lipid nanoparticles coated with nickel ions on the surface for the delivery of the his-Tat cationic antigen against the HIV virus. The authors underline that the most relevant aspect related to the administration of his-Tat/Ni-NPs does not consist in an improvement of the adjuvant activity, compared to the anionic NPs coated with Tat with sodium dodecyl sulfate (SDS/NP), but with less toxicity than that found in dendritic cells. The Ni-NPs were also analyzed for the co-transport of the two protein antigens p24 and Nef. The results obtained by administration to female BALB/c mice showed a greater immune response, making this formulation suitable for the development of multivalent vaccines. Zazo et al. [57] exploited the efficacy of AuNPs as a vector of stavudine, an antiretroviral drug against HIV that targets primary macrophages. AuNPs of various sizes (between 10 and 70 nm) were functionalized with polyethylene glycol (PEG), polyethylene imine (PEI), and citrate, and subsequently linked to stavudine. Comparing the results obtained by administering AuNP-stavudine with those obtained by stavudine alone, the authors noted how the use of NPs induced high variation in terms of morphology of macrophages, with a consequent increase in the efficacy of the drug. To obtain the same results, it was necessary to administer large quantities of stavudine alone, indicating that the use of the carrier allows to obtain a significant increase in intracellular uptake. In fact, the authors hypothesized that the passive absorption of stavudine is usually low due its hydrophilic properties.

Paul et al. [58] synthesized AuNPs linked to interfering RNAs (siRNA) to improve their metabolic stability in the treatment against DENV infection. The AuNPs complexes were encapsulated within a cationic polymeric bilayer in order to increase the conjugation between siRNA and AuNPs. The positive charge of the synthesized cationic complex also allowed to increase the ability to cross the negatively charged cell membrane, significantly inhibiting the replication of DENV-2. Drug delivery systems have found considerable use in recent years, especially due to the onset of the COVID-19 infection. Metal nanoparticles play a key role in the development of safer formulations that are aimed at culling this epidemic disease. Bibi et al. [74] proposed fullerene C60 that was doped with metals (Fe, Cr, and Ni) as a potential agent for the delivery of favipiravir (FPV), a drug that is widely used due to its high efficacy against infection. Through computational studies that were aimed to define the structural and electronic properties in aqueous solution, the authors observed that the doping C60 with Fe, Ni, or Cr significantly improved the rate of FPV administration but also induced greater control of release and a decrease in side effects. The interaction of FPV with the three metal-doped fullerenes (CrC59, FeC59, NiC59) was investigated by determining the binding energy (Eg) and frontier molecular orbital (FMO). The results showed a decrease in Eg following the interaction between FPV and doped C60, which results in higher electronic properties. Furthermore, the authors have pointed out that the FPV-NiC59 complexes showed the highest conductivity value [13].

For the treatment of infectious diseases against the respiratory tract, Zachar [61] proposed the administration of colloidal metal nanoparticles (NpC) for the delivery of various drugs through controlled inhalation of aerosols. In particular, it is underlined the efficacy of NpCs, including silver NpCs, with a size between 2–10 nm and with a high negative zeta potential, is due to a major electrostatic interaction with the spike proteins of generally positively charged viruses.

## 6. Perspectives and Conclusions

This review aims to highlight that the use of drug-metal antiviral complexes and metal-based nanostructured materials has not only allowed to significantly increase the antiviral action of pre-existing drugs but has also led to the formulation of new effective antiviral therapies. To understand the behavior and mechanism of action of these metal complexes, it is essential to define their chemical characteristics, paying particular attention to their thermodynamic and structural properties. Certainly, these aspects are required to be further investigated and more studies are needed to accurately define some thermodynamic properties, such as acid-base behavior and solubility in aqueous solutions, but also some other important characteristics, such as lipophilicity and polymorphism. A greater awareness of some of these properties has also led to the development of new vaccine therapies that are characterized by higher and longer lasting humoral immune responses. Indeed, the use of MeNPs as adjuvant agents has been described to lead to potential benefits in current vaccines and has facilitated the formulation of next-generation ones against various infectious diseases. The development of nanotechnologies has also made it possible to consider MeNPs, in particular AuNP and AgNP, excellent candidates for the transport and controlled release of antiviral drugs, thanks above all to their inert nature and high ability to bind drugs if they are properly functionalized. Although there are several papers attesting to the effectiveness of these systems, others are still in progress and others need to be performed in order to obtain safer and more easily administered formulations.

## Figures and Tables

**Table 1:** Metal complexes and their applications in antiviral therapy.

Metal	Ligand	Application	Virus	Ref.
Au(I)	2,3,4,6-tetra-o-acetyl-L-thio-β-D-glycol-pyranoses-S-(triethyl-phosphine) (AF)	Antiviral therapy	HIV-1, SARS-CoV-2	[8,25,28]
	Aurothiomalate	Antiviral therapy	HIV-1	[25]
	Aurothioglucose	Antiviral therapy	HIV-1	[31]
Ga(III)	Curcumin	Antiviral therapy	HSV-1	[13,42]
Co(III)	Doxovir	Antiviral therapy	HSV-1	[37]
Cu(II) Ni(II) Co(II) Zn(II) Cr(III)	Tenoxicam, valine	Vaccine therapy	Infectious Bovine Rhinotracheitis (IBR)	[48]
	5,5-[3-diyl)]bis(quinolin-8-ole)	Vaccine therapy	Bovine respiratory syncytial (BRS)	[49]
AuNPs	FMD virus-like particles (VLPs)	Vaccine therapy	Foot-and-mouth disease (FMD)	[50]
	West Nile Virus (WNV) envelope (E) protein	Vaccine therapy	West Nile Virus (WNV)	[51]
	STG antigen	Vaccine therapy	Enteropathogenic coronavirus of transmissible porcine gastroenteritis (STG)	[52]
	PEG-S461-493	Vaccine therapy	SARS-CoV-2	[53]
	Protein S	Vaccine therapy	SARS-CoV-2	[54]
	Raltegravir (RAL)	Drug delivery system	HIV	[55]
	FMD virus protein	Drug delivery system	FMD	[56]
	Stavudine	Drug delivery system	HIV	[57]
	siRNA	Drug delivery system	DENV	[58]
Fe(III)	NanoMOFs	Drug delivery system	HIV	[59]
	Tenoxicam, valine	Vaccine therapy	IBR	[48]
NiNPs	His-Tat cationic antigen	Drug delivery system	HIV	[60]
AgNPs	Spike proteins	Drug delivery system	SARS-CoV-2	[61]
